# Physiological Responses of *Syringa oblata* Seedlings to Foliar Salicylic Acid Under Short-Term Heat Stress

**DOI:** 10.3390/biology15161389

**Published:** 2026-08-13

**Authors:** Baolong Du, Weigang Fu, Jinbo Li, Yuan Wang, Juexian Dong, Jinlong Li, Nan Xu, Haixiu Zhong

**Affiliations:** 1 Heilongjiang Province Key Laboratory of Cold Region Wetland Ecology and Environment Research, School of Geography and Tourism, Harbin University, Harbin 150086, China; 2 Heilongjiang Academy of Sciences Institute of Natural Resources and Ecology, Harbin 150040, China

**Keywords:** antioxidant defense, chlorophyll fluorescence, gas exchange, heat stress, OJIP, oxidative injury, photosystem II, salicylic acid, *Syringa oblata*

## Abstract

High temperature can impair leaf water status, photosynthetic function, and membrane stability in ornamental woody seedlings. However, integrated evidence combining gas exchange, chlorophyll fluorescence, oxidative injury, antioxidant activity, and osmotic-adjustment-related responses in heat-stressed *Syringa oblata* remains limited. One-year-old seedlings from a single nursery batch were exposed for 7 d to 25/18 °C or 40/30 °C day/night conditions and sprayed with either a solvent solution or 0.5 mM salicylic acid. Growth, leaf water status, photosynthetic pigments, gas exchange, pulse-amplitude-modulated fluorescence, OJIP/JIP-test parameters, oxidative-injury markers, antioxidant enzyme activities, and osmotic-adjustment-related compounds were evaluated. Heat treatment reduced leaf relative water content, photosynthetic performance, and photosystem II function and increased reactive oxygen species accumulation, lipid peroxidation, and electrolyte leakage. Compared with heat treatment alone, seedlings receiving salicylic acid under heat showed an 82.2% higher net photosynthetic rate, a 12.2% higher maximum quantum efficiency of photosystem II, and a 116.4% higher performance index on an absorption basis. Hydrogen peroxide, malondialdehyde, and electrolyte leakage were 35.1%, 35.8%, and 32.5% lower, respectively. Antioxidant enzyme activities were also higher under heat plus salicylic acid than under heat alone, whereas additional increases in proline and soluble sugars were not statistically confirmed; soluble protein was partially maintained. Gas exchange was measured after treatment at a common leaf-chamber temperature of 25 °C and therefore represented retained photosynthetic capacity under standardized conditions. Overall, foliar application of 0.5 mM salicylic acid was associated with partial maintenance of photosynthetic function and lower oxidative injury during short-term heat exposure. Because one chamber was assigned to each treatment combination in a single experimental run, possible chamber-specific effects could not be statistically separated from treatment-related differences. Independent validation is therefore required.

## Simple Summary

High temperatures can reduce the growth and ornamental quality of lilac seedlings by disturbing leaf water balance, photosynthesis, and membrane stability. This study examined whether spraying salicylic acid could reduce short-term heat injury in *Syringa oblata*. Heat-treated seedlings showed visible leaf injury, lower water content, reduced photosynthetic activity, and greater membrane damage. Seedlings receiving salicylic acid during heat treatment retained more leaf water, showed better photosynthetic performance, and had lower oxidative injury than seedlings exposed to heat alone. These findings provide an integrated physiological description of the short-term response of lilac seedlings to salicylic acid under controlled heat-stress conditions. Because each treatment was assigned to one chamber in a single controlled-environment run, possible chamber-specific effects could not be separated from treatment-related differences. Independently replicated chamber, greenhouse, and field studies are therefore required before the generality or practical application of these responses can be evaluated.

## 1. Introduction

High temperature can restrict the establishment and ornamental performance of woody seedlings by disturbing leaf water status, photosynthetic carbon assimilation, photosystem II (PSII) function, and cellular redox balance. Reduced stomatal conductance, pigment loss, impaired electron transport, and excessive reactive oxygen species accumulation may occur concurrently during heat exposure, contributing to membrane lipid peroxidation, electrolyte leakage, visible leaf injury, and reduced growth [1,2,3]. Gas exchange and pulse-amplitude-modulated chlorophyll fluorescence provide complementary information on carbon assimilation and PSII photochemistry, whereas OJIP transients and JIP-test parameters can further characterize stress-associated changes in reaction-center performance, electron transport, and energy dissipation [3,4].

*Syringa oblata* Lindl. is a temperate woody ornamental species valued for its spring flowering and landscape use. Previous studies have shown that leaf gas exchange and chlorophyll fluorescence in *Syringa* species respond to environmental stress [5,6]. However, the short-term heat response of *S. oblata* seedlings has not been comprehensively characterized across water status, standardized post-treatment gas-exchange capacity, PAM fluorescence, OJIP/JIP-test performance, oxidative injury, antioxidant enzyme activity, and osmotic-adjustment-related compounds. Integrating these measurements may provide a more complete physiological description than evaluating individual endpoints separately.

Salicylic acid is an endogenous phenolic signaling molecule involved in plant responses to environmental stress [7]. Recent studies have associated exogenous SA application with changes in photosynthetic performance, antioxidant enzyme activities, membrane stability, and stress-related metabolites under elevated temperature. In bell pepper, SA treatments improved antioxidant and osmotic responses under high-temperature conditions [8]. SA-associated maintenance of photosynthetic pigments and modulation of antioxidant responses have also been reported in heat-stressed cotton and fenugreek [9,10], whereas foliar SA improved chlorophyll-fluorescence-related performance in heat-stressed apple leaves [11]. In heat-stressed grapevines, SA application was associated with higher photosynthetic performance, improved PSII photochemical efficiency, lower lipid peroxidation, and greater soluble protein content [12]. Related changes in water status, chlorophyll fluorescence, oxidative injury, and antioxidant enzyme activities have also been reported in the woody species *Pterocarya fraxinifolia* under drought and cadmium stress [13]. Collectively, these studies indicate that SA-associated responses vary with plant species, concentration, developmental stage, application method, and stress intensity and duration.

The present study therefore evaluated the short-term physiological and biochemical responses of *S. oblata* seedlings to foliar application of 0.5 mM SA under controlled heat conditions. The contribution of the study lies in combining growth, leaf water status, photosynthetic pigments, standardized gas exchange, PAM fluorescence, OJIP/JIP-test parameters, oxidative-injury markers, antioxidant enzyme activities, and osmotic-adjustment-related compounds within the same experimental framework. We tested whether HT+SA seedlings would show less pronounced heat-associated injury and better maintenance of photosynthetic and membrane-related traits than HT seedlings. The experiment was designed to characterize an integrated endpoint response after 7 d rather than to establish an optimal SA dose, a temporal sequence, or a molecular mechanism.

## 2. Materials and Methods

### 2.1. Plant Material and Growth Conditions

One-year-old nursery-grown *Syringa oblata* Lindl. seedlings were obtained from Xiangda Nursery, Xiangfang District, Harbin, Heilongjiang Province, China. All seedlings used in the experiment originated from the same nursery batch. Detailed provenance and propagation information was not recorded at the time of plant acquisition. Seedlings were selected to have a comparable developmental stage, similar shoot architecture, fully expanded and visually healthy leaves, and no visible symptoms of disease, insect injury, mechanical damage, severe chlorosis, or abnormal growth. At the beginning of acclimation, the selected seedlings were approximately 25 cm in height and had a stem diameter of approximately 3 mm.

Each plant was transplanted individually into a plastic pot with a top diameter of approximately 18 cm, a bottom diameter of 14 cm, and a height of 18 cm. Each pot was filled with approximately 3.0 L of a sterilized peat–perlite–vermiculite substrate mixed at a volume ratio of 3:1:1.

The plants were acclimated for 30 d in the Plant Physiology Laboratory, College of Life Sciences, Northeast Forestry University, Harbin, China. The subsequent 7 d of temperature and salicylic acid treatments were conducted in four controlled-environment chambers at the Ecological Conservation Laboratory, Institute of Natural Resources and Ecology, Heilongjiang Academy of Sciences, Harbin, China. During acclimation, the plants were maintained under a 14 h/10 h light/dark photoperiod, day/night temperatures of 25/18 °C, and a relative humidity of 65–75%. Illumination was provided by the built-in lighting system of the controlled-environment chamber (Mobaite, Hangzhou, Zhejiang, China; model MB100). The photosynthetic photon flux density at plant-canopy height was maintained at approximately 250 μmol m^−2^ s^−1^. The spectral distribution of the light source was not independently measured during the experiment; therefore, wavelength-specific spectral characteristics and the relative contributions of individual spectral regions are not reported.

During acclimation and the 7 d treatment period, all pots were irrigated once every 7 d. At each irrigation, water was applied slowly until the first appearance of drainage from the bottom of the pot. The same operational endpoint and irrigation schedule were used for all pots. The exact irrigation volume and substrate water content were not recorded. Plants were repositioned periodically during acclimation to reduce positional effects. Fully expanded leaves at comparable developmental positions were used for physiological measurements and biochemical analyses.

### 2.2. Experimental Design and Treatments

The experiment was conducted in four controlled-environment chambers at the Ecological Conservation Laboratory, Institute of Natural Resources and Ecology, Heilongjiang Academy of Sciences, Harbin, China. The four chambers were operated simultaneously, and one chamber was assigned to each treatment combination.

A concentration of 0.5 mM was selected as a physiologically relevant test concentration based on previous studies in which the same or comparable foliar salicylic acid concentrations produced measurable physiological responses under heat and other abiotic stresses, including studies involving woody plants [8,11,13]. The present experiment was designed to evaluate the response of *Syringa oblata* seedlings to one defined salicylic acid concentration rather than to determine an optimal dose.

The 7 d treatment period was used to characterize short-term physiological and biochemical responses to heat exposure. All physiological and biochemical measurements were conducted at the predefined endpoint. Accordingly, the experiment was not designed to determine the onset or temporal sequence of the observed responses.

Four treatments were established: normal temperature with solvent spray (CK), normal temperature with 0.5 mM salicylic acid (SA), high temperature with solvent spray (HT), and high temperature with 0.5 mM salicylic acid (HT+SA). The prescribed temperature regimes were 25/18 °C day/night for CK and SA and 40/30 °C day/night for HT and HT+SA. Relative humidity, substrate composition, irrigation schedule and watering criterion, and treatment duration were maintained as consistently as possible among chambers, except for the prescribed temperature and SA treatments. The prescribed 25/18 °C and 40/30 °C day/night regimes refer to chamber air-temperature settings. Leaf temperature during the 7 d exposure period was not independently monitored using thermocouples or thermal imaging.

Analytical-grade salicylic acid (SA; 99.9% purity) was purchased from Sigma-Aldrich (St. Louis, MO, USA). SA was first dissolved in a small volume of ethanol and then diluted with distilled water to a final concentration of 0.5 mM. The final ethanol concentration was below 0.1% (*v*/*v*), and Tween-20 was added as a surfactant at a final concentration of 0.05% (*v*/*v*). Plants in the CK and HT treatments received the same solvent solution without SA. The SA or solvent solution was sprayed uniformly onto both the adaxial and abaxial leaf surfaces until runoff at 24 h before the start of the temperature treatment and again on Day 3.

Each treatment chamber contained six independently potted seedlings, with one seedling per pot. These seedlings were treated as plant-level subsamples within the corresponding treatment chamber rather than as independent chamber-level treatment replicates. Pots were randomly positioned and repositioned daily to reduce within-chamber positional variation. The four chambers were operated simultaneously, and non-target environmental settings were maintained as consistently as possible among chambers. Nevertheless, because one chamber was assigned to each treatment combination, independent chamber-level replication was not included.

### 2.3. Phenotype Evaluation, Growth Traits, and Leaf Water Status

Representative plants were photographed after the 7 d treatment to document visible heat-injury symptoms. Visual heat injury was evaluated using a six-level ordinal scale: 0, no visible injury; 1, very slight leaf curling or loss of turgor; 2, mild wilting or chlorosis affecting a limited portion of the foliage; 3, moderate wilting, curling, or chlorosis affecting multiple leaves; 4, severe wilting or chlorosis accompanied by localized necrosis; and 5, extensive wilting, chlorosis, and/or necrosis affecting most of the foliage. Because the score was ordinal, it was analyzed using a non-parametric procedure. Visual injury was assessed by the same trained evaluator using the predefined criteria. Treatment identities were not formally masked during the original assessment; this potential source of subjectivity is acknowledged as a study limitation.

Plant height was measured from the substrate surface to the shoot apex using a ruler. Stem diameter was measured using a digital caliper. Leaf area was measured using an LI-3000C portable leaf area meter (LI-COR Biosciences, Lincoln, NE, USA). Shoots were harvested and weighed immediately to obtain shoot fresh weight. Samples were then heated at 105 °C for 30 min and dried at 75 °C to constant weight to determine shoot dry weight.

Leaf relative water content was measured using the relative turgidity method described by Barrs and Weatherley [14]. Fresh leaf disks were weighed immediately after sampling to obtain fresh weight (FW), floated in distilled water for 6 h in darkness to obtain turgid weight (TW), and then dried at 75 °C to constant weight to obtain dry weight (DW). Relative water content was calculated as follows: RWC (%) = (FW − DW)/(TW − DW) × 100.

### 2.4. Photosynthetic Pigments and SPAD Values

The SPAD values were obtained by measurement with a SPAD-502 Plus chlorophyll meter (Konica Minolta, Tokyo, Japan). For each plant-level subsample, five SPAD readings were taken from fully expanded leaves while avoiding the main vein, and their mean was used for analysis.

Photosynthetic pigments were extracted from 0.20 g of fresh leaf tissue in 20 mL of 80% acetone in darkness until the tissue was completely decolorized. The extract was centrifuged at 10,000× *g* for 10 min. Absorbance at 663, 646, and 470 nm was measured using a UV-1800 UV–visible spectrophotometer (Shimadzu, Kyoto, Japan). Chlorophyll a, chlorophyll b, and carotenoid contents were calculated according to Lichtenthaler [15] and expressed as mg g^−1^ FW.

### 2.5. Gas-Exchange Measurements

Gas-exchange parameters were measured between 09:00 and 11:30 using a CIRAS-3 portable photosynthesis system (PP Systems, Amesbury, MA, USA). Fully expanded mature leaves at comparable developmental positions were used. The leaf-chamber conditions were as follows: photosynthetic photon flux density, 1000 μmol m^−2^ s^−1^; CO_2_ concentration, 400 μmol mol^−1^; leaf temperature, 25 °C; and relative humidity, 60–70%.

Stabilized net photosynthetic rate (P*_n_*), stomatal conductance (G*_s_*), intercellular CO_2_ concentration (C*_i_*) and transpiration rate (T*_r_*) were measured. Instantaneous water-use efficiency was determined using the following equation: WUE = P*_n_*/T*_r_*.

where P_n_ is given in units of μmol CO_2_ m^−2^ s^−1^ and T_r_ is given in units of mmol H_2_O m^−2^ s^−1^. Gas-exchange measurement and interpretation were in line with the general methodological concerns discussed on measurement and analysis of gas-exchange in photosynthesis [16].

All gas-exchange measurements were conducted after the 7 d treatment at a common cuvette leaf temperature of 25 °C. Accordingly, these measurements represented the photosynthetic capacity retained by the leaves under standardized measurement conditions rather than instantaneous gas exchange during exposure to the 40 °C daytime treatment temperature.

### 2.6. Chlorophyll Fluorescence Measurements

Chlorophyll fluorescence parameters were measured using an FMS-2 pulse-modulated chlorophyll fluorometer (Hansatech Instruments Ltd., King’s Lynn, UK). Leaves were dark-adapted for 30 min using leaf clips before measurement. Minimum fluorescence (F_o_) was recorded under weak measuring light, and maximum fluorescence (F_m_) was obtained using a saturating pulse. The maximum quantum efficiency of PSII was calculated as: F_v_/F_m_ = (F_m_ − F_o_)/F_m_.

After dark-adapted measurements, leaves were exposed to actinic light for light adaptation. Steady-state fluorescence (F_s_) and maximum fluorescence under light (F_m_′) were recorded. The effective quantum yield of PSII [Y_(*II*)_], electron transport rate (ETR), non-photochemical quenching (NPQ), and photochemical quenching coefficient (qP) were calculated according to standard chlorophyll fluorescence protocols [17,18,19]. ETR was calculated as: ETR = Y_(*II*)_ × PPFD × 0.84 × 0.5.

where PPFD was the actinic light intensity during fluorescence measurement, 0.84 represents the assumed leaf absorptance, and 0.5 represents the assumed equal distribution of absorbed light energy between PSI and PSII.

### 2.7. OJIP Transient and JIP-Test Analysis

Fast chlorophyll a fluorescence transients were recorded using a Handy PEA chlorophyll fluorometer (Hansatech Instruments Ltd., King’s Lynn, UK). Leaves were dark-adapted for 30 min before measurement. Fluorescence induction curves were recorded under a saturating red light pulse for 1 s.

The OJIP transient was used to evaluate PSII photochemical performance under heat stress and salicylic acid treatment. Relative variable fluorescence at the J step (V*_J_*) and the K-step-related parameter (W*_K_*) were calculated from the fluorescence transient. JIP-test parameters, including ABS/RC, ET_o_/RC, DI_o_/RC, and PI*_ABS_*, were calculated according to the JIP-test framework [4]. ABS/RC represents absorbed energy flux per active reaction center; ET_o_/RC represents electron transport flux per active reaction center; DI_o_/RC represents dissipated energy flux per active reaction center; and PIABS represents the performance index on an absorption basis. Heat-induced alterations of PSII donor-side and acceptor-side performance, energy absorption, electron transport and dissipation of energy were measured by OJIP and JIP-test parameters.

### 2.8. Oxidative Damage and Antioxidant Enzyme Activities

After the physiological measurements, fresh leaf samples were immediately collected, frozen in liquid nitrogen, and stored at −80 °C until biochemical analysis. For enzyme extraction, 0.50 g of fresh leaf tissue was homogenized with 5 mL of ice-cold 50 mM potassium phosphate buffer (pH 7.0) containing 1 mM EDTA and 1% (*w*/*v*) polyvinylpolypyrrolidone. The homogenate was centrifuged at 12,000× *g* and 4 °C for 15 min, and the supernatant was used to determine antioxidant enzyme activities.

Hydrogen peroxide content was determined using the titanium (IV) method [20] and expressed as μmol g^−1^ FW. The O_2_•^−^ production rate was determined using the hydroxylamine method [21] and expressed as nmol g^−1^ FW min^−1^. Malondialdehyde content was determined using the thiobarbituric acid method [22] and expressed as nmol g^−1^ FW. Relative electrolyte leakage was measured using a DDS-307A conductivity meter (Leici, Shanghai, China). Leaves were immersed in deionized water for 12 h, after which the initial conductivity (C_1_) was recorded. The samples were then boiled for 20 min, cooled to room temperature, and used to determine the final conductivity (C_2_). Relative electrolyte leakage was calculated as follows: REL (%) = C_1_/C_2_ × 100.

The superoxide dismutase activity was estimated by the extent of suppression of nitroblue tetrazolium photoreduction at 560 nm [23]. The peroxidase activity was measured with the guaiacol-H_2_O_2_ method by recording the rise in absorbance at 470 nm [24]. Catalase activity was measured by observing the reduction of H_2_O_2_ at 240 nm [25]. Ascorbate peroxidase activity was quantified by determining the reduction in absorbance at 290 nm caused by oxidation of ascorbate [26]. Enzyme activities were reported in U g^−1^ FW.

### 2.9. Osmotic Adjustment Substances

The acid ninhydrin method was used to determine free proline content [27]. Fresh leaves were ground in 3% sulfosalicylic acid and boiled in a water bath. Following centrifugation, acid ninhydrin reagent and glacial acetic acid were added to the supernatant and incubated at 100 °C. The reaction mixture was subjected to toluene extraction and absorbance measured at 520 nm. Proline content was quantified by a standard curve and reported in terms of u mol g^−1^ FW.

The soluble sugar content was determined by the anthrone-sulfuric acid procedure [28]. The fresh leaf tissue was boiled in distilled water. Anthrone reagent and concentrated sulfuric acid were used to react with the extract and absorbance was determined at 620 nm. The content of soluble sugars was reported as mg g^−1^ FW.

The concentration of soluble proteins was assayed by the methodology of Coomassie Brilliant Blue G-250 [29]. Phosphate buffer was used to extract the fresh leaf tissue, which was then combined with Coomassie Brilliant Blue reagent. The absorbance was read at 595 nm and the standard protein used was bovine serum albumin. Soluble protein content was reported as mg g^−1^ FW.

### 2.10. Statistical Analysis

Data are presented as means ± standard deviations based on six plant-level subsamples within each treatment chamber. Statistical analyses were performed using SPSS 26.0 (IBM Corp., Armonk, NY, USA). These analyses describe differences among plant-level subsamples in the four treatment groups within the present controlled-environment run. Because independent chamber-level replication was not included, treatment-related differences cannot be statistically separated from possible chamber-specific effects, and the results should not be interpreted as chamber-replicated estimates of treatment effects.

Differences among CK, SA, HT, and HT+SA were evaluated using one-way analysis of variance followed by Tukey’s honestly significant difference test. Normality of model residuals and homogeneity of variance were examined using the Shapiro–Wilk and Levene tests, respectively. Welch’s analysis of variance was used when the assumption of homogeneity of variance was not met. Statistical significance was defined as *p* < 0.05.

Visual injury scores were treated as ordinal data and analyzed using the Kruskal–Wallis test followed by Dunn’s multiple-comparison procedure. Derived variables, including water-use efficiency and fluorescence ratios, were calculated separately for each plant before statistical analysis. Different lowercase letters in the figures indicate significant differences among the four treatment combinations.

The measured variables represented predefined physiological endpoints in distinct functional categories and were analyzed separately. No global multiple-testing correction was applied across all measured endpoints. Accordingly, interpretation focused primarily on response patterns that were consistent across related physiological categories, whereas isolated, marginal, or non-significant comparisons were treated cautiously.

## 3. Results

### 3.1. Effects of Salicylic Acid on Visual Injury, Growth Traits, and Leaf Water Status Under Heat Stress

**Figure 1:**
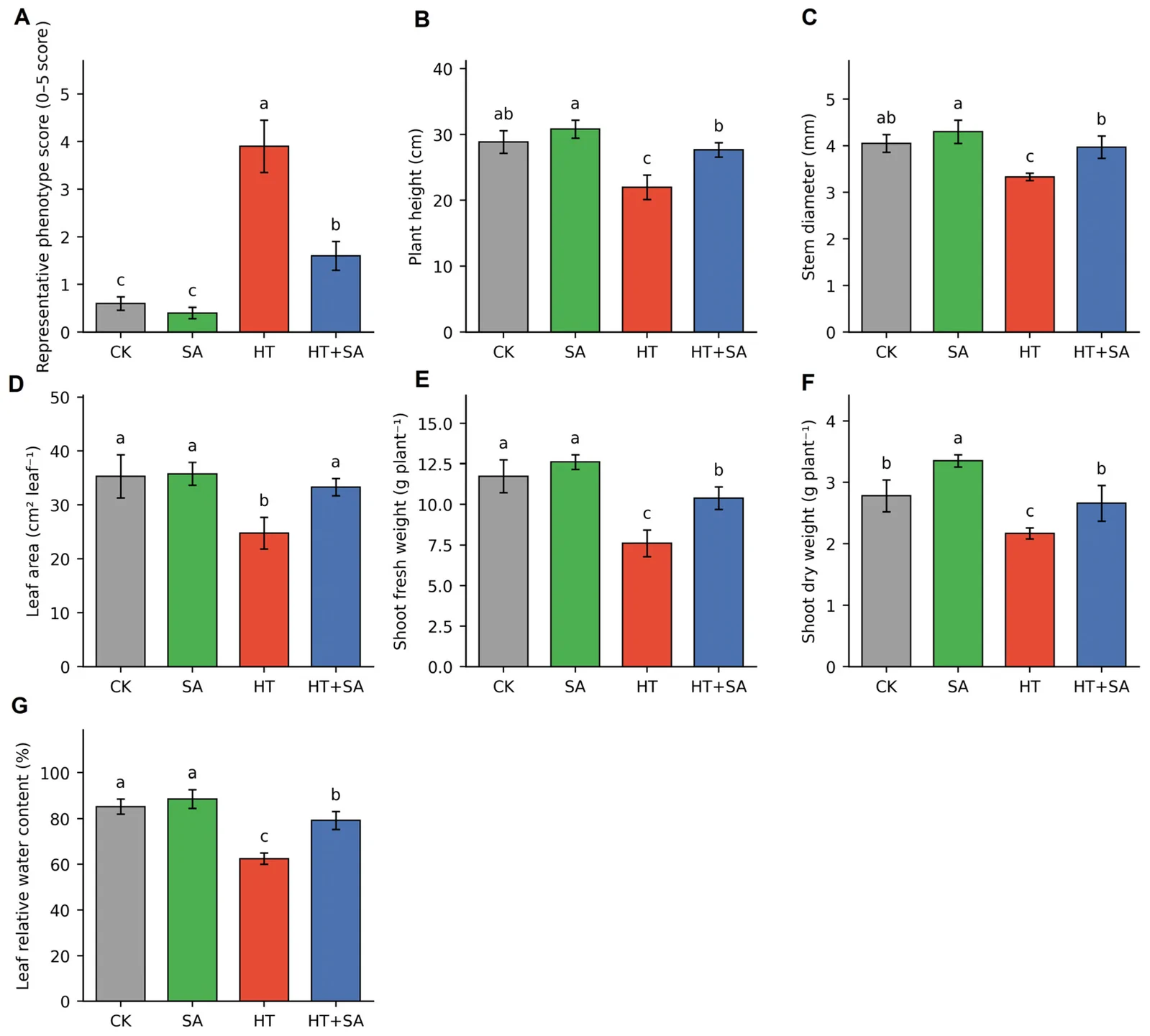
Effects of salicylic acid on phenotype, growth traits, and leaf water status of *Syringa oblata* seedlings under heat stress. Effects of different treatments on (**A**) visual injury score, (**B**) plant height, (**C**) stem diameter, (**D**) leaf area, (**E**) shoot fresh weight, (**F**) shoot dry weight, and (**G**) leaf relative water content. CK, normal temperature plus solvent spray; SA, normal temperature plus 0.5 mM salicylic acid; HT, heat stress plus solvent spray; HT+SA, heat stress plus 0.5 mM salicylic acid. Values are means ± SD based on six plant-level subsamples within each treatment chamber. Different lowercase letters indicate significant differences among treatments at *p* < 0.05. Visual injury score was analyzed using the Kruskal–Wallis test followed by Dunn’s multiple-comparison test; other variables were analyzed using Tukey’s HSD test.

Heat treatment increased the visual injury score from 0.6 in CK to 3.9 in HT, whereas the score in HT+SA was 1.6 (Figure 1A). Compared with CK, HT decreased plant height, stem diameter, leaf area, shoot fresh weight, shoot dry weight, and leaf relative water content by 23.8%, 17.8%, 29.8%, 35.2%, 21.9%, and 26.7%, respectively (Figure 1B–G). Compared with HT, HT+SA increased these variables by 25.7%, 19.2%, 34.5%, 36.5%, 22.6%, and 26.7%, respectively.

### 3.2. Effects of Salicylic Acid on Photosynthetic Pigments and Gas Exchange Under Heat Stress

**Figure 2:**
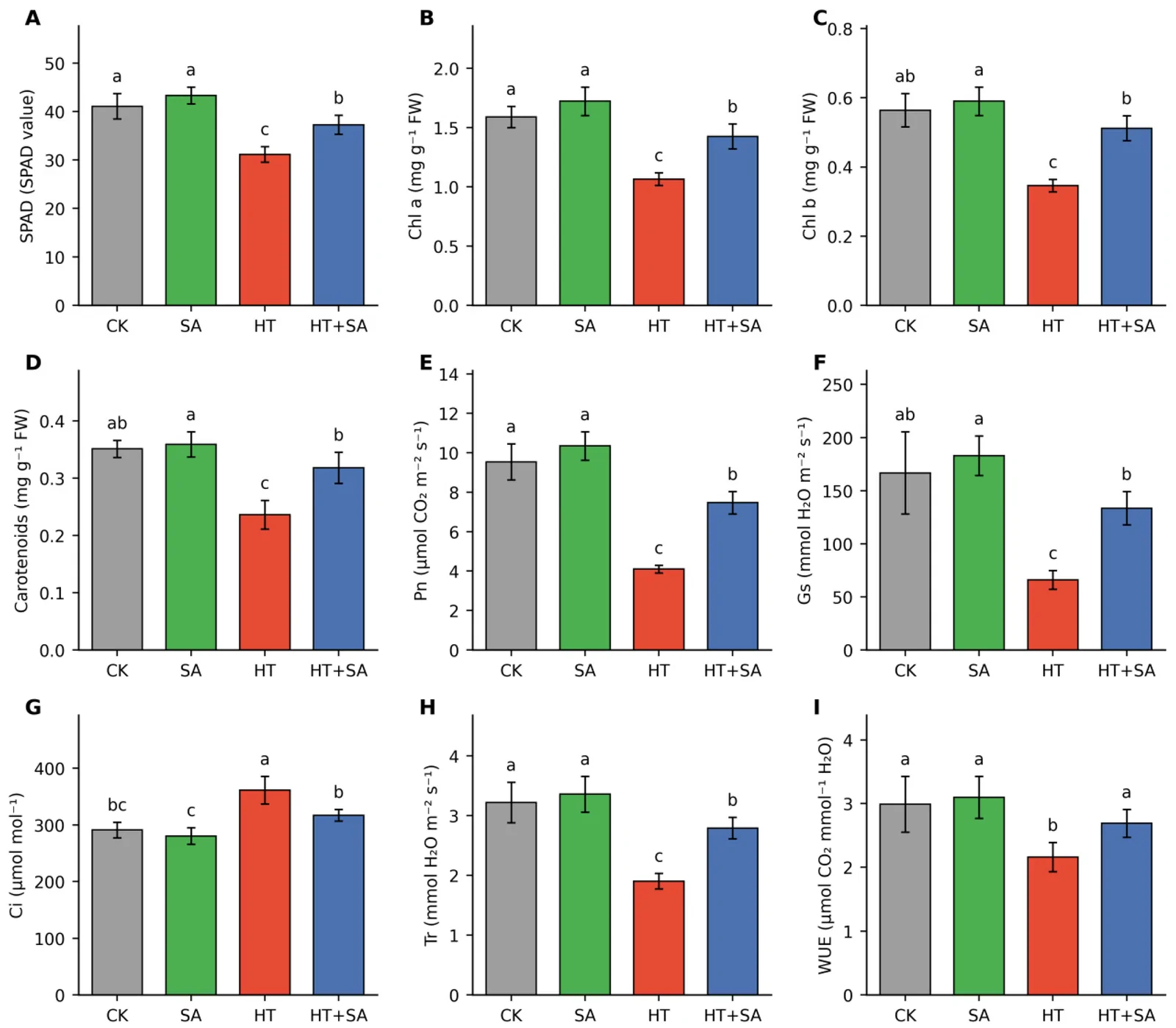
Effects of salicylic acid on photosynthetic pigments and gas-exchange parameters in *Syringa oblata* leaves under heat stress. Effects of different treatments on (**A**) SPAD value, (**B**) chlorophyll a, (**C**) chlorophyll b, (**D**) carotenoids, (**E**) net photosynthetic rate (P*_n_*), (**F**) stomatal conductance (G*_s_*), (**G**) intercellular CO_2_ concentration (C*_i_*), (**H**) transpiration rate (T*_r_*), and (**I**) instantaneous water-use efficiency (WUE). Values are means ± SD based on six plant-level subsamples within each treatment chamber. Different lowercase letters indicate significant differences among treatments according to Tukey’s HSD test at *p* < 0.05.

Compared with CK, HT decreased SPAD value, chlorophyll a, chlorophyll b, and carotenoid contents by 24.2%, 33.0%, 38.7%, and 32.8%, respectively (Figure 2A–D). Compared with HT, HT+SA increased these variables by 19.6%, 33.8%, 48.0%, and 34.7%, respectively.

Compared with CK, HT decreased net photosynthetic rate (P*_n_*), stomatal conductance (G*_s_*), transpiration rate (T*_r_*), and instantaneous water-use efficiency (WUE) by 57.0%, 60.5%, 41.0%, and 27.8%, respectively, and increased intercellular CO_2_ concentration (C*_i_*) by 24.2% (Figure 2E–I). Compared with HT, HT+SA increased P*_n_*, G*_s_*, T*_r_*, and WUE by 82.2%, 102.4%, 46.8%, and 24.5%, respectively, and decreased C*_i_* by 12.3%. These measurements were obtained at the common leaf-chamber temperature of 25 °C.

### 3.3. Effects of Salicylic Acid on PSII Photochemical Parameters Under Heat Stress

**Figure 3:**
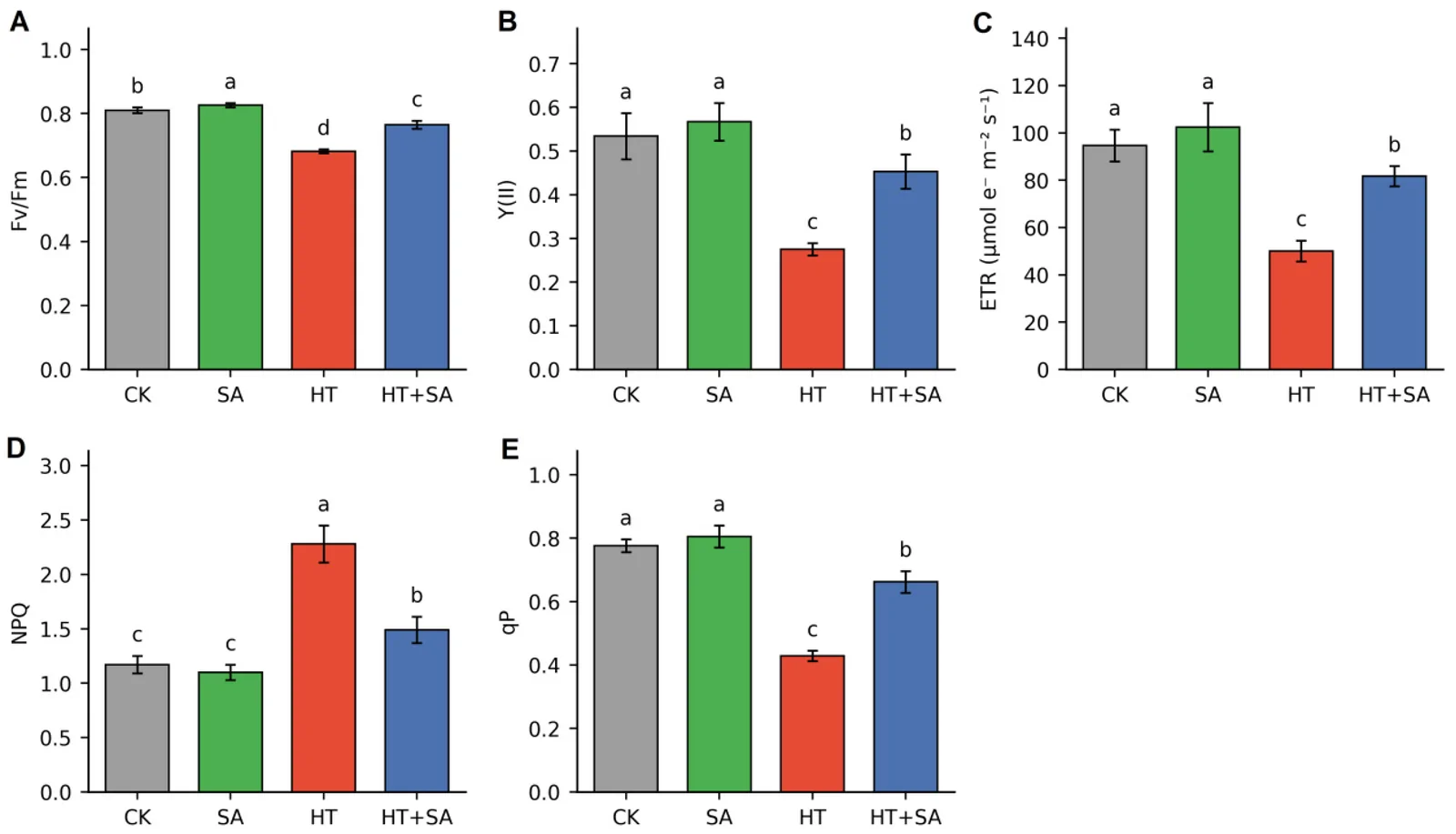
Effects of salicylic acid on FMS-2 chlorophyll fluorescence parameters in *Syringa oblata* leaves under heat stress. Effects of different treatments on (**A**) F_v_/F_m_, (**B**) Y_(II)_, (**C**) electron transport rate (ETR), (**D**) non-photochemical quenching (NPQ), and (**E**) photochemical quenching coefficient (qP). Values are means ± SD based on six plant-level subsamples within each treatment chamber. Different lowercase letters indicate significant differences among treatments according to Tukey’s HSD test at *p* < 0.05.

Compared with CK, HT decreased F*_v_*/F*_m_*, Y_(*II*)_, electron transport rate (ETR), and photochemical quenching coefficient (qP) by 15.8%, 48.5%, 47.1%, and 44.7%, respectively, and increased non-photochemical quenching (NPQ) by 94.9% (Figure 3A–E). Compared with HT, HT+SA increased F_v_/F_m_, Y_(II)_, ETR, and qP by 12.2%, 64.7%, 63.1%, and 54.3%, respectively, and decreased NPQ by 34.6%.

### 3.4. Effects of Salicylic Acid on OJIP-Derived JIP-Test Parameters Under Heat Stress

**Figure 4:**
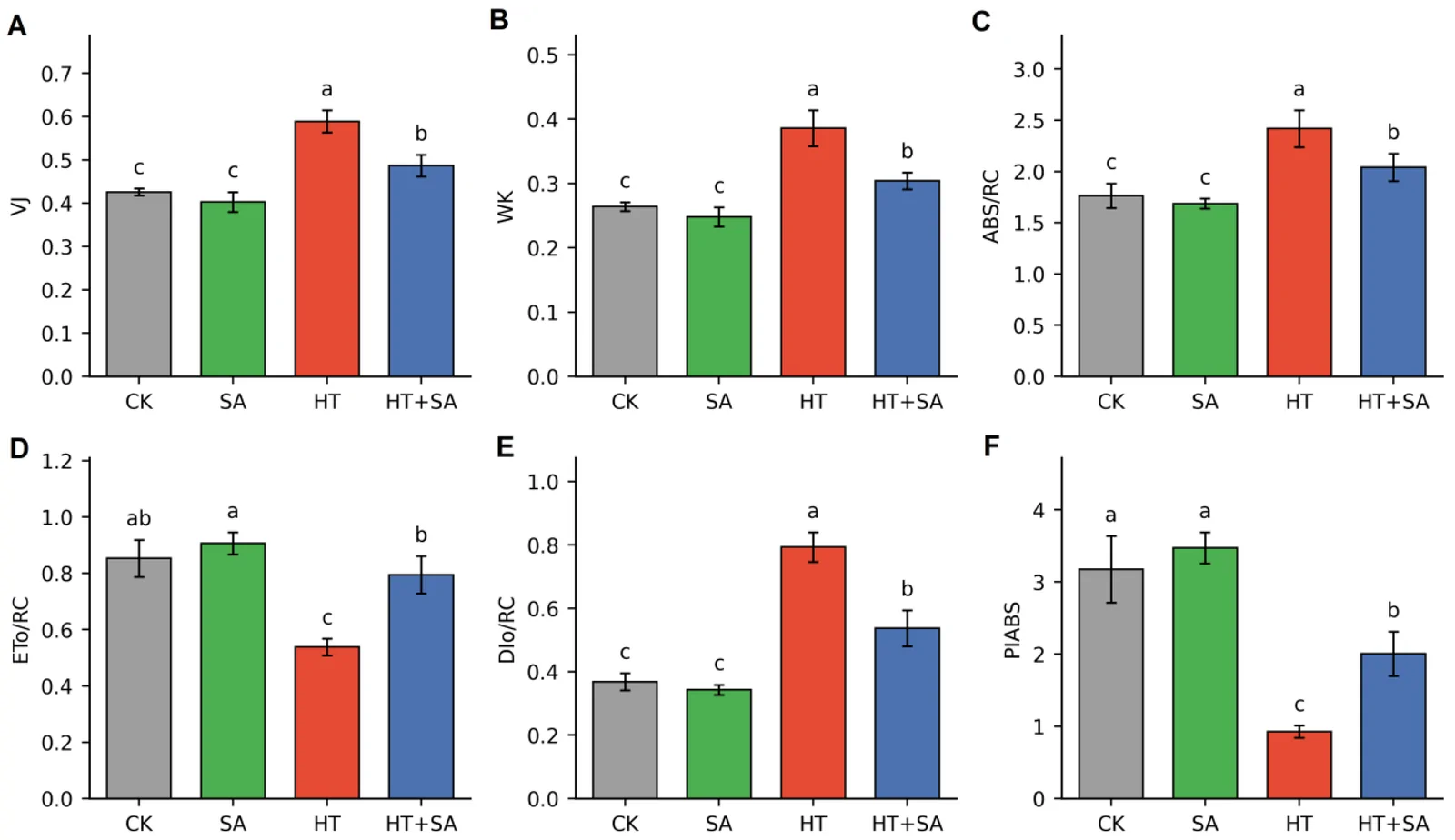
Effects of salicylic acid on Handy PEA OJIP-derived JIP-test parameters in *Syringa oblata* leaves under heat stress. Effects of different treatments on (**A**) V*_J_*, (**B**) W*_K_*, (**C**) ABS/RC, (**D**) ET_o_/RC, (**E**) DI_o_/RC, and (**F**) PI*_ABS_*. V*_J_* indicates relative variable fluorescence at the J step; WK indicates K-step-related fluorescence change; ABS/RC, ET_o_/RC, and DI_o_/RC represent absorbed energy flux, electron transport flux, and dissipated energy flux per active PSII reaction center, respectively; PI*_ABS_* represents the performance index on an absorption basis. Values are means ± SD based on six plant-level subsamples within each treatment chamber. Different lowercase letters indicate significant differences among treatments according to Tukey’s HSD test at *p* < 0.05.

Compared with CK, HT increased V*_J_*, W*_K_*, ABS/RC, and DI_o_/RC by 38.3%, 46.2%, 37.2%, and 115.5%, respectively, and decreased ET_o_/RC and PIABS by 36.9% and 70.8%, respectively (Figure 4A–F). Compared with HT, HT+SA decreased V*_J_*, W*_K_*, ABS/RC, and DI_o_/RC by 17.3%, 21.2%, 15.6%, and 32.3%, respectively, and increased ET_o_/RC and PIABS by 47.8% and 116.4%, respectively.

### 3.5. Effects of Salicylic Acid on Oxidative-Injury Markers Under Heat Stress

**Figure 5:**
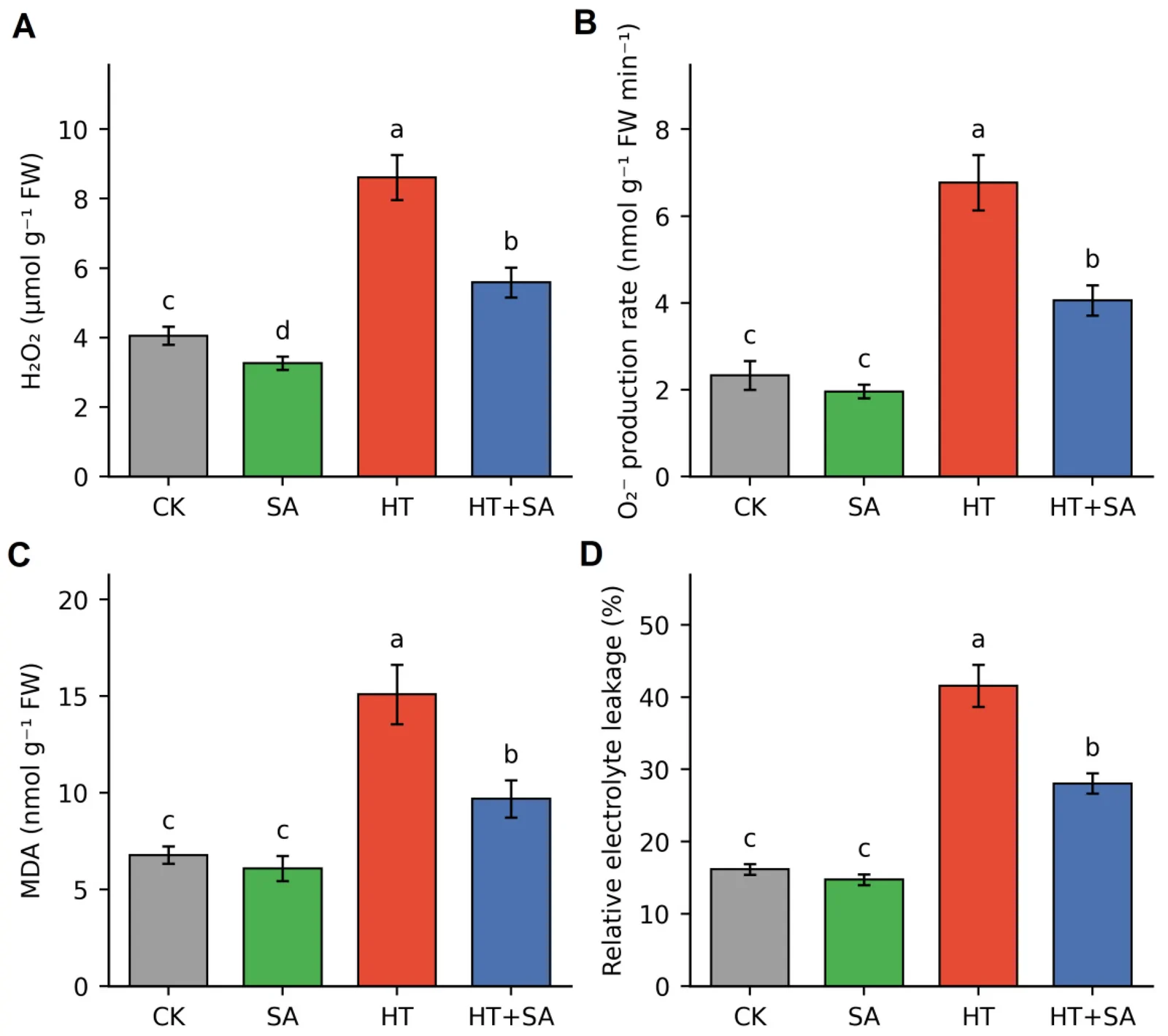
Effects of salicylic acid on ROS accumulation and membrane injury in *Syringa oblata* leaves under heat stress. Effects of different treatments on (**A**) H_2_O_2_ content, (**B**) O_2_^−^ production rate, (**C**) malondialdehyde (MDA) content, and (**D**) relative electrolyte leakage. Values are means ± SD based on six plant-level subsamples within each treatment chamber. Different lowercase letters indicate significant differences among treatments according to Tukey’s HSD test at *p* < 0.05.

Compared with CK, HT increased H_2_O_2_ content, O_2_•^−^ production rate, malondialdehyde content, and relative electrolyte leakage by 112.6%, 190.6%, 122.6%, and 157.2%, respectively (Figure 5A–D). Compared with HT, HT+SA decreased these variables by 35.1%, 40.0%, 35.8%, and 32.5%, respectively. The values in HT+SA generally remained higher than those in CK and SA.

### 3.6. Effects of Salicylic Acid on Antioxidant Enzyme Activities and Osmotic-Adjustment-Related Compounds Under Heat Stress

**Figure 6:**
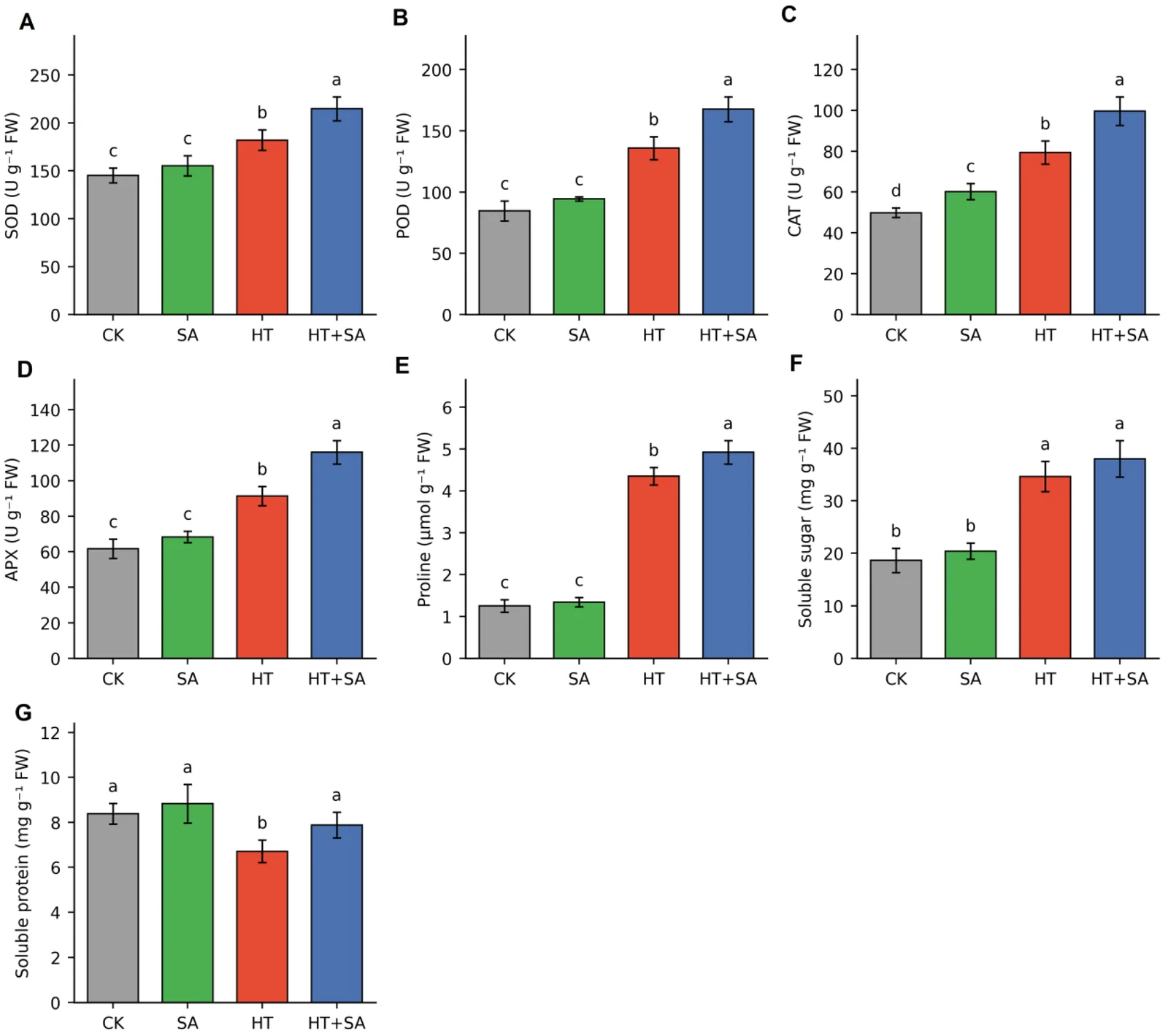
Effects of salicylic acid on antioxidant enzyme activities and osmotic adjustment substances in *Syringa oblata* leaves under heat stress. Effects of different treatments on (**A**) SOD activity, (**B**) POD activity, (**C**) CAT activity, (**D**) APX activity, (**E**) proline content, (**F**) soluble sugar content, and (**G**) soluble protein content. Values are means ± SD based on six plant-level subsamples within each treatment chamber. Different lowercase letters indicate significant differences among treatments according to Tukey’s HSD test at *p* < 0.05.

Compared with CK, HT increased SOD, POD, CAT, and APX activities by 25.4%, 60.7%, 59.2%, and 48.0%, respectively (Figure 6A–D). Compared with HT, HT+SA increased the activities of these enzymes by 18.0%, 23.3%, 25.6%, and 27.1%, respectively.

Compared with CK, HT increased proline and soluble sugar contents by 248.0% and 85.7%, respectively, and decreased soluble protein content by 19.9% (Figure 6E–G). Proline and soluble sugar contents were numerically higher in HT+SA than in HT, but the differences were not statistically significant. In contrast, soluble protein content was significantly higher in HT+SA than in HT.

## 4. Discussion

### 4.1. Water Status, Growth, and Standardized Post-Treatment Gas-Exchange Capacity

The lower leaf relative water content, leaf area, and shoot biomass observed in HT were consistent with a short-term heat-associated reduction in plant water status and final growth-related values. In woody seedlings, reduced water status can constrain cell expansion and leaf development and may occur together with decreased carbon assimilation during heat exposure. The higher leaf relative water content and biomass-related values in HT+SA than in HT indicate that the combined treatment was associated with less pronounced water-status and growth reduction under the present conditions.

Gas exchange was measured after the treatment at a common leaf-chamber temperature of 25 °C. Consequently, the lower P*_n_*, G*_s_*, T***_r_***, and WUE observed in HT represented a reduction in photosynthetic capacity retained after the 7 d treatment rather than instantaneous gas exchange at 40 °C. The concurrent higher Pn and Gs and lower C*_i_* in HT+SA indicated that these seedlings retained a greater capacity for CO_2_ assimilation under standardized measurement conditions. These measurements do not establish that SA increased instantaneous photosynthesis during heat exposure or restored a specific stomatal regulatory mechanism.

The present pattern is broadly consistent with recent reports of SA-associated maintenance of water status, growth, or photosynthetic performance under elevated temperature in bell pepper, cotton, fenugreek, apple, and grapevine [8,9,10,11,12,30,31,32,33]. Nevertheless, the magnitude of these responses varies among species, concentrations, application methods, and treatment durations.

### 4.2. Photosynthetic Pigments and PSII Photochemical Responses

The decreases in SPAD value, chlorophyll contents, and carotenoids under HT were consistent with reduced light-harvesting and photoprotective capacity after short-term heat exposure. In HT+SA, the higher pigment values than in HT coincided with better maintenance of the measured photosynthetic traits. Similar SA-associated pigment responses have recently been reported in heat-stressed cotton and other species [9].

The PAM-fluorescence measurements further showed lower F_v_/F_m_, Y_(*II*)_, ETR, and qP and higher NPQ in HT. This combination indicates lower PSII photochemical performance and a greater relative allocation of absorbed excitation energy to non-photochemical dissipation. The higher F_v_/F_m_, Y_(*II*)_, ETR, and qP and lower NPQ in HT+SA were consistent with partial maintenance of PSII-related performance. Recent work in heat-stressed apple leaves similarly reported changes in chlorophyll-fluorescence-related traits following foliar SA treatment [11,33,34].

Although the decrease in F_v_/F_m_ suggests heat-associated PSII inhibition, fluorescence variables alone cannot distinguish all structural and regulatory processes contributing to this response. The results therefore support functional differences in PSII performance rather than direct evidence of structural repair.

### 4.3. OJIP/JIP-Test Responses and PSII Reaction-Center Performance

OJIP-derived indices provided information complementary to the PAM-fluorescence measurements. The concurrent increases in V*_J_* and W*_K_* under HT were consistent with restrictions in electron transport beyond Q*_A_* and possible impairment of PSII donor-side function. In particular, an increase in W*_K_* may be associated with altered donor-side or oxygen-evolving-complex performance. However, these variables are indirect functional indicators and cannot independently demonstrate structural damage to the oxygen-evolving complex.

The increase in ABS/RC and DI_o_/RC, together with decreases in ET_o_/RC and PI*_ABS_*, indicated a shift toward greater apparent energy absorption and dissipation per active reaction center and lower electron-transport performance. The opposite changes in HT+SA were consistent with partial maintenance of PSII reaction-center performance. The agreement between PAM and OJIP-derived variables strengthens the interpretation that the two measurement systems detected a coherent PSII-related response pattern, but it does not establish the underlying molecular process [3,4].

### 4.4. Oxidative Injury and Antioxidant Enzyme Responses

The higher H_2_O_2_ content, O_2_•^−^ production rate, MDA content, and relative electrolyte leakage in HT collectively indicated greater reactive oxygen species accumulation, lipid peroxidation, and membrane permeability. The lower values of all four injury-related variables in HT+SA than in HT showed that the combined treatment coincided with less pronounced oxidative and membrane injury, although the values generally remained above those under normal-temperature conditions.

SOD, POD, CAT, and APX activities were higher in HT+SA than in HT. SOD participates in O_2_•^−^ conversion, whereas POD, CAT, and APX contribute to H_2_O_2_ metabolism. Thus, the concurrent occurrence of higher antioxidant enzyme activities and lower oxidative-injury markers is consistent with an association between antioxidant responses and lower injury. Recent heat-stress studies in cotton and fenugreek have also reported SA-associated changes in antioxidant enzyme activity and oxidative-injury markers [9,10].

The current data do not show how SA altered enzyme activity. Changes could involve transcriptional, translational, post-translational, metabolic, or substrate-related processes, none of which were directly measured. Endogenous SA was also not quantified. The results therefore describe coordinated biochemical responses rather than a confirmed SA-regulated ROS-scavenging mechanism.

### 4.5. Partial Maintenance of Soluble Protein, with Limited Evidence for Additional Osmolyte Accumulation

Heat treatment increased proline and soluble sugar contents, which is consistent with a general stress-associated osmotic and metabolic response. However, neither variable differed significantly between HT and HT+SA. The present results therefore do not demonstrate that SA further increased proline or soluble sugar accumulation beyond the response induced by heat alone.

In contrast, soluble protein content was lower in HT than in CK and was partially maintained in HT+SA. This pattern may reflect better maintenance of protein-related metabolic status or lower cellular injury in HT+SA seedlings. Because protein synthesis, degradation, and denaturation were not directly evaluated, the result should be interpreted as an association with soluble-protein maintenance rather than evidence of a specific protein-protection mechanism.

### 4.6. Integrated Physiological Responses and Study Limitations

The measured variables showed a coherent pattern across photosynthetic function, oxidative injury, and antioxidant activity. Compared with HT, HT+SA seedlings had higher Pn, Fv/Fm, Y(II), ET_0_/RC, and PIABS and lower H_2_O_2_, MDA, and electrolyte leakage. Higher antioxidant enzyme activities also occurred concurrently with lower oxidative-injury markers. Together, these changes support an integrated physiological response pattern in which better maintenance of gas-exchange and PSII-related traits coincided with less pronounced oxidative and membrane injury. However, the endpoint measurements do not establish the temporal sequence or direction of causality among these responses.

The experiment was conducted as a single controlled-environment run, with one chamber assigned to each treatment combination. Although the chambers were operated simultaneously under matched non-target settings and the independently potted seedlings were repositioned daily, independent chamber-level replication was not included. Consequently, possible chamber-specific effects cannot be statistically separated from treatment-related differences. The findings should therefore be interpreted as plant-level response patterns observed within the present experimental run rather than as definitive chamber-replicated estimates of treatment effects.

The plant material originated from one nursery batch, and the study did not compare different provenances, genotypes, propagation sources, or developmental stages. Only one salicylic acid concentration and a 7 d exposure period were evaluated. The study therefore did not establish a dose–response relationship, identify an optimal concentration, evaluate longer-term acclimation, or determine the temporal development of the responses.

The prescribed temperature regimes referred to chamber air temperature. Actual leaf temperature during the treatment period was not independently monitored, and possible treatment-related differences in leaf-to-air temperature could not be evaluated. Gas exchange was measured after treatment at a standardized leaf temperature of 25 °C and represented retained photosynthetic capacity rather than instantaneous photosynthesis at the treatment temperature.

The study did not quantify endogenous salicylic acid, gene expression, protein abundance, or targeted metabolic pathways. Consequently, the observed changes cannot identify the molecular processes underlying the response to foliar salicylic acid. In addition, multiple physiological endpoints were analyzed separately without a global correction across all measured variables, which may increase the cumulative probability of Type I error. The interpretation therefore emphasizes coherent response patterns across related measurements and does not rely on isolated or non-significant comparisons.

Independent chamber repetitions, dose- and time-response experiments, different seedling sources, direct leaf-temperature monitoring, and greenhouse and field validation will be required to assess the generality and practical relevance of the present response pattern.

## 5. Conclusions

Short-term exposure to 40/30 °C day/night conditions was associated with visible leaf injury, lower leaf water status and final growth-related values, reduced standardized post-treatment gas-exchange capacity, lower PSII-related performance, and greater oxidative and membrane injury in *Syringa oblata* seedlings. Compared with HT, HT+SA seedlings showed better maintenance of photosynthetic pigments, gas-exchange and PSII-related traits, lower reactive oxygen species accumulation, lipid peroxidation, and electrolyte leakage, and higher antioxidant enzyme activities. Among the measured osmotic-adjustment-related compounds, the clearest SA-associated response was the partial maintenance of soluble protein; proline and soluble sugar contents were not significantly higher in HT+SA than in HT.

Overall, foliar application of 0.5 mM SA was associated with an integrated pattern of partial photosynthetic maintenance and lower oxidative injury under the present short-term controlled conditions. The study was limited to one nursery batch, one species, one developmental stage, one SA concentration, and one 7 d experimental run in which each treatment was assigned to one chamber. Gas exchange was measured at a standardized leaf temperature of 25 °C, and actual leaf temperature during heat exposure was not independently monitored. The results therefore do not establish an optimal SA concentration, a temporal or molecular mechanism, or a practical nursery recommendation. Independent chamber repetitions, dose- and time-response experiments, and greenhouse and field validation are required before broader application can be considered.

## Data Availability

The original contributions presented in the study are included in the article; further inquiries can be directed to the corresponding author.
